# A Head-to-head Comparison of Prostate Cancer Diagnostic Strategies Using the Stockholm3 Test, Magnetic Resonance Imaging, and Swedish National Guidelines: Results from a Prospective Population-based Screening Study

**DOI:** 10.1016/j.euros.2022.01.010

**Published:** 2022-02-18

**Authors:** Mauritz Waldén, Mattias Aldrimer, Jakob Heydorn Lagerlöf, Martin Eklund, Henrik Grönberg, Tobias Nordström, Thorgerdur Palsdottir

**Affiliations:** a Department of Urology, Central Hospital of Karlstad, Karlstad, Sweden; b Department of Clinical Chemistry, Central Hospital of Karlstad, Karlstad, Sweden; c Department of Medical Physics, Central Hospital of Karlstad, Karlstad, Sweden; d Department of Medical Physics, Faculty of Medicine and Health, Örebro University, Örebro, Sweden; e Department of Medical Epidemiology and Biostatistics, Karolinska Institutet, Solna, Sweden; f Department of Clinical Sciences, Danderyd Hospital, Danderyd, Sweden

**Keywords:** Prostate cancer, Prostate neoplasm, Prostate cancer screening, Cancer screening, Stockholm3, Magnetic resonance imaging

## Abstract

**Background:**

Strategies for early detection of prostate cancer aim to detect clinically significant prostate cancer (csPCa) and avoid detection of insignificant cancers and unnecessary biopsies. Swedish national guidelines (SNGs), years 2019 and 2020, involve prostate-specific antigen (PSA) testing, clinical variables, and magnetic resonance imaging (MRI). The Stockholm3 test and MRI have been suggested to improve selection of men for prostate biopsy. Performance of SNGs compared with the Stockholm3 test or MRI in a screening setting is unclear.

**Objective:**

To compare strategies based on previous and current national guidelines, Stockholm3, and MRI to select patients for biopsy in a screening-by-invitation setting.

**Design, setting, and participants:**

All participants underwent PSA test, and men with PSA ≥3 ng/ml underwent Stockholm3 testing and MRI. Men with Stockholm3 ≥11%, Prostate Imaging Reporting and Data System score ≥3 on MRI, or indication according to SNG-2019 or SNG-2020 were referred to biopsy.

**Outcome measurements and statistical analysis:**

The primary outcome was the detection of csPCa at prostate biopsy, defined as an International Society of Urological Pathology (ISUP) grade of ≥2.

**Results and limitations:**

We invited 8764 men from the general population, 272 of whom had PSA ≥3 ng/ml. The median PSA was 4.1 (interquartile range: 3.4–5.8), and 136 of 270 (50%) who underwent MRI lacked any pathological lesions. In total, 37 csPCa cases were diagnosed. Using SNG-2019, 36 csPCa cases with a high biopsy rate (179 of 272) were detected and 49 were diagnosed with ISUP 1 cancers. The Stockholm3 strategy diagnosed 32 csPCa cases, with 89 biopsied and 27 ISUP 1 cancers. SNG-2020 detected 32 csPCa and 33 ISUP 1 cancer patients, with 99 men biopsied, and the MRI strategy detected 30 csPCa and 35 ISUP 1 cancer cases by biopsying 123 men. The latter two strategies generated more MRI scans than the Stockholm3 strategy (*n* = 270 vs 33).

**Conclusions:**

Previous guidelines provide high detection of significant cancer but at high biopsy rates and detection of insignificant cancer. The Stockholm3 test may improve diagnostic precision compared with the current guidelines or using only MRI.

**Patient summary:**

The Stockholm3 test facilitates detection of significant cancer, and reduces the number of biopsies and detection of insignificant cancer.

## 1 Introduction

Prostate cancer screening studies show reduced prostate cancer mortality with prostate-specific antigen (PSA) testing followed by systematic biopsy for men with elevated PSA levels [1], [2]. However, organized population-based prostate cancer screening has not been introduced in any country except Lithuania and Kazakhstan, due to the risk of overdiagnosis and overtreatment [3].

To improve prostate cancer diagnostics, risk prediction models using clinical variables and biomarkers have been developed. These models, for example, Stockholm3, Prostate Cancer Prevention Trial Risk Calculator, Prostate Biopsy Collaborative Group Risk Calculator, European Randomised Study of Screening for Prostate Cancer, 4K, and Prostate Health Index, have shown favorable results compared with PSA to detect clinically significant prostate cancer (csPCa) [4], [5], [6], [7], [8], [9]; at the same time, these have been shown to reduce the number of biopsies performed and International Society of Urological Pathology (ISUP) grade 1 cancers diagnosed [10]. Studies using prebiopsy magnetic resonance imaging (MRI) have increased biopsy precision [11], [12] and reduced the number of low-risk cancers detected [13], which has led to recommendations to include MRI in diagnostic algorithms [14], [15], [16].

In Sweden, from 2007 until 2020, five versions of the Swedish national prostate cancer guidelines (SNGs) have been published [17], [18]. These guidelines recommend the combined use of clinical variables, biomarkers, and imaging to refer men to prostate biopsy. Version 4 (introduced in 2019 and valid between 2019 and 2020 [SNG-2019]) recommended the use of age, clinical T stage, PSA, free PSA, and PSA density for selecting men to undergo biopsy. They also define recommendations for repeat biopsies with the use of MRI. Version 5 (introduced in 2020 [SNG-2020]) combines previous guidelines with the use of MRI to recommend men to undergo biopsy. The guidelines have not been validated for detecting csPCa in a screening setting.

The aim of this study was to compare the precision of the Stockholm3 test or MRI with contemporary national guidelines in Sweden to select patients for the detection of csPCa in a screening setting.

## 2 Patients and methods

### 2.1 Participants and study design

**Fig. 1:**
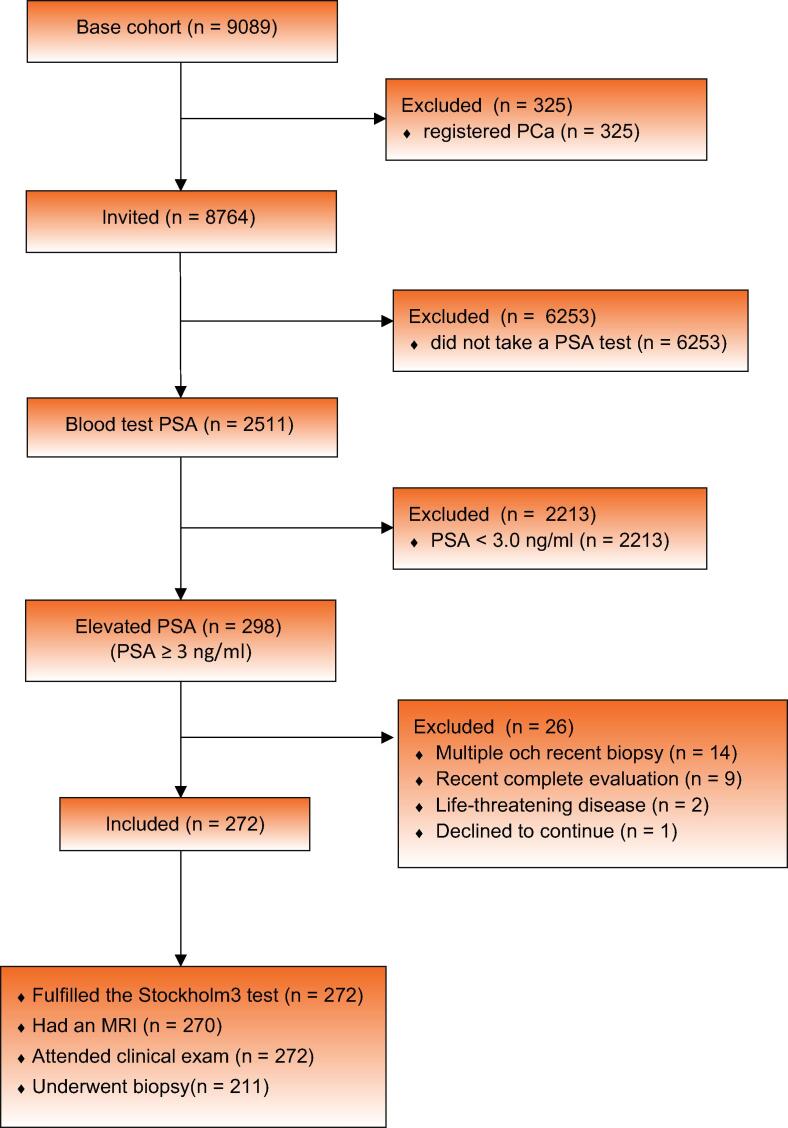
Consort flow diagram of the Stockholm3 versus SNG study. MRI = magnetic resonance imaging; PCa = prostate cancer; PSA = prostate-specific antigen; SNG = Swedish national guideline.

The Stockholm3 versus SNG study was a population-based, paired, screening-by-invitation study inviting all men without a previous diagnosis of prostate cancer, born in 1950, 1954, 1959, 1965, and 1970 in Värmland County, Sweden. Patients were recruited from September 2019 until May 2020, and participants underwent blood sampling and a PSA test. Those with PSA ≥3 ng/ml underwent a Stockholm3 test and MRI of the prostate (Fig. 1). Men with an increased risk of prostate cancer according to the national guidelines 2019 had a ten-core systematic biopsy. Men with no indication according to the national guidelines but positive Stockolm3 had a ten-core biopsy or those with a Prostate Imaging Reporting and Data System (PI-RADS) score of ≥ 3 on MRI had targeted biopsies to complete three parallel strategies. Men with a benign biopsy result or ISUP grade 1 prostate cancer on the first biopsy underwent a targeted repeat biopsy if they had PI-RADS ≥3 on MRI or a systematic repeat biopsy after 3 mo if there was an indication of increased risk according to any of the guidelines.

The study was approved by the Ethics Review Authority (Dnr 2019-00104).

### 2.2 Stockholm 3 test

The Stockholm3 test predicts a man’s risk of having csPCa in biopsy, based on a combination of plasma protein biomarkers (PSA, free PSA, hK2, MSMB, and MIC1), genetic markers, and clinical information (age, family history, prostate volume, and previous prostate biopsy).

### 2.3 MRI and biopsy procedures

MRI was performed in all cases using Siemens Magnetom Skyra^fit^, 3 Tesla, with body coil and integrated back coil. The acquisition time was 22 min, and the protocol consisted of a T2-weighted Half-Fourier Acquisition Single-shot Turbo spin Echo (HASTE) sequence followed by an axial T1-weighted turbo spin echo (tse), a T2-weighted tse in all directions, and a diffusion-weighted sequence with *b* values of 50, 500, 1000 (measured), and 1500 (calculated) s/mm^2^. Sequence parameters are described in Supplementary Table 1. MRI investigations were read by any of ten external experienced uroradiologists at the telemedicine clinic (https://www.telemedicineclinic.com) using PI-RADS v 2.1, and for all cases with PI-RADS ≥3, a second evaluation was performed by one reader in a high-volume center—the Radiological Clinic of the Örebro University Hospital.

Systematic transrectal ultrasound (TRUS)-guided ten-core dorsal biopsies were performed under local anesthesia after oral antibiotic prophylaxis. In systematic repeat biopsies, four ventral cores were added. MRI fusion using the Koelis system targeted two to three biopsies of each focus. In seven cases, cognitive biplane TRUS-guided targeted biopsies were performed. The ISUP 2014 criteria for Gleason scoring was applied by 15 pathologists in two centralized centers in Karlstad and Örebro [19].

### 2.4 Evaluated strategies

We compared four different screening strategies, two SNGs (SNG-2019 and SNG-2020 version), a strategy based on the Stockholm3 test, and a strategy using only MRI to identify men to be subjected to biopsy.

**Fig. 2:**
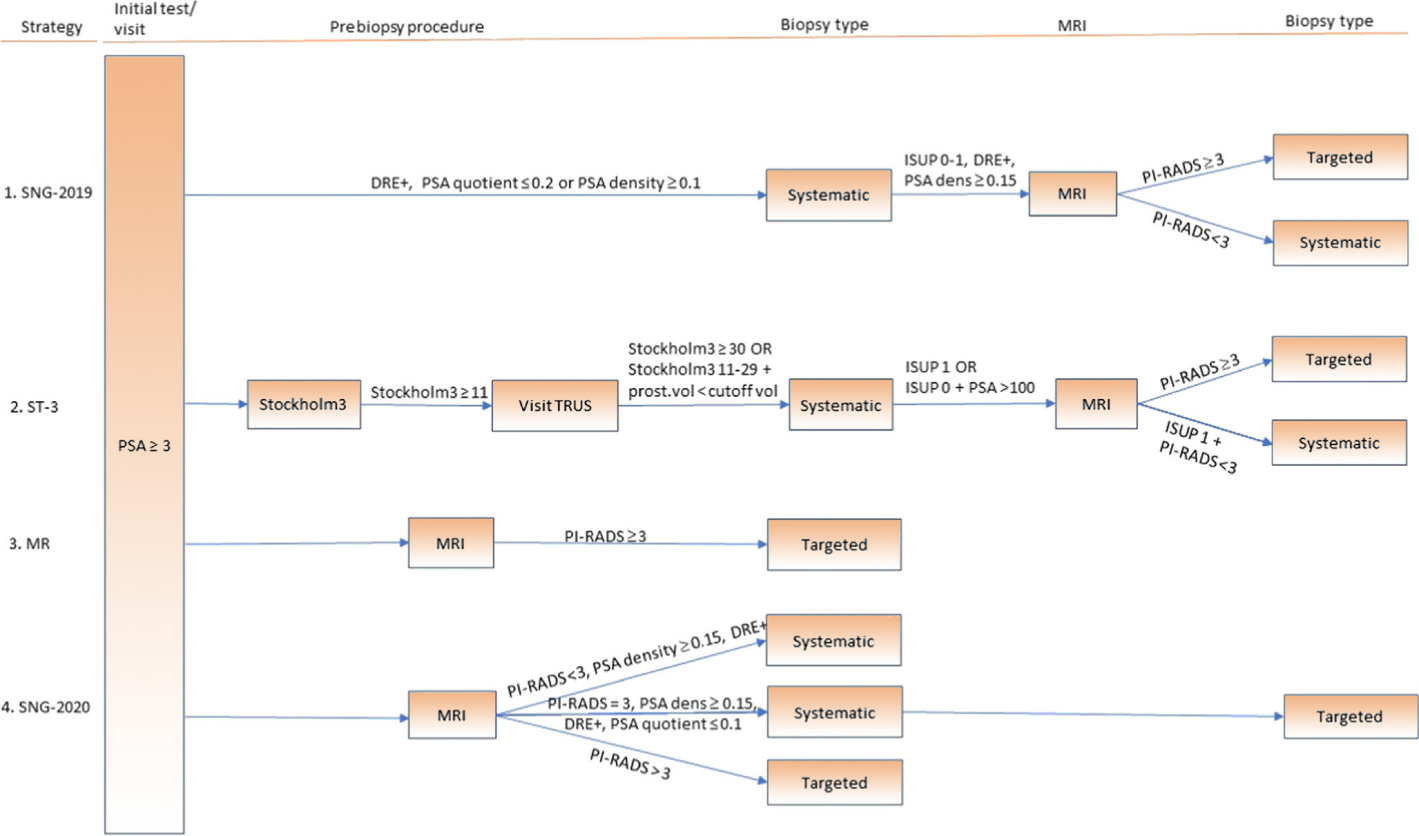
Clinical flow of different diagnostic strategies in the Stockholm3-SNG study. SNG-2019 represents previous Swedish national guidelines for screening for prostate cancer without MRI and SNG-2020 represents current Swedish national guidelines including MRI. In the paired screening by invitation study design, all men underwent a PSA test and those with PSA ≥3 ng/ml underwent a Stockholm3 test and MRI. If men were at a higher risk by indication from any of the four different screening strategies, they were referred to continue in the clinical diagnostic process according to the respective strategy. For example, in the SNG-2020 strategy, a man with PSA ≥3 ng/ml first undergoes MRI. If the MRI result is PI-RADS 3 and if either PSA density ≥0.15 or DRE is positive or PSA quotient ≤0.1, the man undergoes a systematic and targeted biopsy. DRE = digital rectal examination; ISUP = International Society of Urological Pathology; MR = MRI strategy; MRI = magnetic resonance imaging; PI-RADS = Prostate Imaging Reporting and Data System; prost.vol = prostate volume; PSA = prostate-specific antigen; PSA dens = PSA density; SNG = Swedish national guideline; ST-3 = Stockholm3 strategy; TRUS = transrectal ultrasound.

Following is a description of each strategy (for a more detailed description; see Fig. 2).

#### 2.4.1 SNGs in use in 2019

Participants with a PSA level of ≥3 ng/ml were referred to a urologist, where prostate volume was measured by TRUS and digital rectal examination (DRE) was performed. If DRE was positive, PSA was >20 ng/ml, the quotient (PSA free/PSA total) was ≤0.2, or PSA density (PSA total/prostate volume) was ≥0.1, the participants underwent a systematic ten-core biopsy. Men with a previous negative biopsy had a second ten- to 14-core systematic biopsy if PSA density was ≥0.15. Men with either ISUP grade 1 in the first biopsy or positive DRE or PSA density ≥0.15 had a repeat biopsy, those with a PI-RADS ≥3 on the MRI had a targeted biopsy, and those with PI-RADS <3 had a ten- to 14-core systematic biopsy.

#### 2.4.2 Stockholm3 strategy

Participants with PSA ≥3 ng/ml underwent a Stockholm3 test; if the Stockholm3 score was ≥11%, participants were referred to a urologist to measure prostate volume and perform a DRE. Men with a positive DRE, a Stockholm3 score of ≥30% or a Stockholm3 score between 11% and 29%, and prostate volume below the cutoff volume given by the Stockholm3 test underwent a ten-core systematic biopsy.

If clinically clear prostate cancer (PSA >100 ng/ml) or ISUP 1 cancer was detected on first biopsy, men underwent MRI and a targeted biopsy for PI-RADS ≥3 or a 14-core biopsy for PI-RADS <3. The Stockholm3 strategy (ST-3) included only men selected by the Stockholm3 test.

#### 2.4.3 MRI strategy

Participants with PSA ≥3 ng/ml underwent MRI, and those with PI-RADS ≥3 underwent targeted biopsies directed to each described MRI lesion. Men with a contraindication for MRI had a systematic biopsy on clinical indication. The MRI strategy (MR) included only men with PI-RADS 3–5 on MRI.

#### 2.4.4 SNGs introduced in 2020

The participants with PSA level ≥3 ng/ml underwent MRI. Based on the MRI result, men with PI-RADS <3 or with contraindication for MRI but PSA density ≥0.15 or a positive DRE underwent a systematic biopsy. Men with PI-RADS 3 and PSA density ≥0.15, or a positive DRE or PSA quotient ≤0.1 underwent systematic and targeted biopsies. Men with PI-RADS >3 underwent a targeted biopsy.

### 2.5 Statistical analysis

To compare the four strategies, we counted the number of procedures (indicated for biopsy, performed biopsies, MRI procedures, and biopsy outcomes) for all four strategies. We then calculated the relative proportions (RPs) of these procedures for the different strategies using one diagnostic strategy relative to the reference strategy (SNG-2019). Confidence intervals (CIs) are two-sided 95% empirical bootstrap intervals based on 1000 bootstrap samples. We used R statistical software v.4.0.3 (R Foundation for Statistical Computing, Vienna, Austria) for all analyses.

## 3 Results

Characteristics of the study population are shown in Table 1. The median age of the study participants was 65 yr (interquartile range [IQR]: 60–69) and the median PSA level was 4.1 ng/ml. The median Stockholm3 scores were 12% (IQR: 8–21) for the total study population and 35% (IQR: 20–46) for the men diagnosed with csPCa, while it was 9% (IQR: 7–13) for men with benign biopsy. Of the men in our study population, 50% had a PI-RADS score of ≤2 (136 men), 73 men (27%) had a PI-RADS score of 3, and 61 men (22%) had a PI-RADS score of ≥4. In total, 211 men had a biopsy, and 37 csPCa and 59 ISUP 1 cases were diagnosed.

**Table 1:** Patient characteristics for 272 men with PSA ≥3 ng/ml in the Stockholm3 versus SNG study between 2019 and 2020.

Variable	All *N* (% column)
All	272 (100)
Age	
49–54	31 (11)
55–59	21 (8)
60–64	55 (20)
65–70	165 (61)
Median (IQR)	65 (60, 69)
Stockholm3	
<11%	118 (43)
11–29%	118 (43)
With indication a	55 (47)
Without indication b	63 (53)
≥30%	36 (13)
Median (IQR)	12 (8, 21)
PSA (ng/ml)	
3–4.9	179 (66)
5–9.9	80 (29)
10–19.9	6 (2)
≥20	7 (3)
Median (IQR)	4.1 (3.4, 5.8)
PSA density (ng/ml/cc)	
<0.1	148 (55)
0.1–0.14	70 (25)
0.15–0.19	24 (9)
≥0.20	30 (11)
Median (IQR)	0.09 (0.06, 0.13)
Previous negative biopsy	
Yes	26 (10)
No	246 (90)
Prostate volume (ml; TRUS)	
<35	66 (24)
35–50	82 (30)
≥50	124 (46)
Median (IQR)	48 (35, 63)
PI-RADS score	
1–2	136 (50)
3	73 (27)
4–5	61 (22)
Missing	2 (1)

IQR = interquartile range; PI-RADS = Prostate Imaging Reporting and Data System; PSA = prostate specific antigen; SNG = Swedish national guidelines; TRUS = transrectal ultrasound.

a With indication: prostate volume on TRUS < cutoff volume.

b Without indication: prostate volume on TRUS ≥ cutoff volume.

### 3.1 Cancer detection using four different diagnostic strategies

**Fig. 3:**
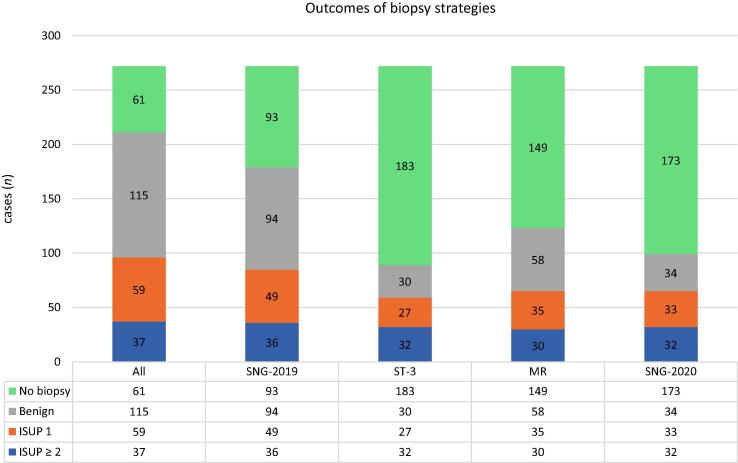
Outcomes of different diagnostic strategies in terms of the number of spared biopsies, and benign, ISUP 1, and ISUP ≥ 2 cancer in biopsy. ISUP = International Society of Urological Pathology; MR = magnetic resonance imaging strategy; SNG = Swedish national guideline; ST-3 = Stockholm3 strategy.

Figure 3 shows the outcome of the different strategies. By selecting men for biopsy using SNG-2019, the total number of biopsy procedures was 179 (65% of total biopsies), while detecting 36 of 37 csPCa (97%) and 49 of 59 ISUP 1 prostate cancer (83%) cases. By using the ST-3 strategy, 89 men were biopsied (33% of total biopsies), while detecting 32 csPCa (86%) and 27 ISUP 1 cancer (46%) cases. With the MR strategy, 123 men underwent biopsy (58%); 30 of 37 csPCa cases were detected (81%) and 35 men were diagnosed with ISUP 1 cancer (59%). The new guidelines SNG-2020 resulted in a decrease in the number of biopsies performed in the total cohort from 211 to 99 (36%), while detecting 32 csPCa (86%) and 33 ISUP 1 cancer (56%) cases. The number of MRI examinations differed for each strategy. In the total study population, all men except two had an MRI scan (270 out of 272). With SNG-2020 and MR strategy, all 270 men underwent MRI; with SNG-2019, 71 men underwent MRI (26%); and with the ST-3 strategy, 33 men underwent MRI (12%), while detecting the same number of csPCa as SNG-2020 (32 out of 37).

### 3.2 RPs of procedures and outcomes of the different strategies

Table 2 shows the number of procedures and RPs of using the ST-3, MR, and SNG-2020 strategies compared with the SNG-2019 strategy. The ST-3 strategy was associated with almost half of the number of biopsies compared with those using SNG-2019 (89 vs 179; RP 0.50 [95% CI 0.41–0.56]), while detecting slightly fewer csPCa cases (32 vs 36; RP 0.89 [95% CI 0.78–0.98]). Comparing SNG-2020 with SNG-2019, the results were similar to those using the ST-3 strategy—a decrease in the number of biopsies (99 vs 179; RP 0.55 [95% CI 0.49–0.65] while detecting slightly fewer csPCa cases (32 vs 36; RP 0.89 [95% CI 0.78–0.98]).

**Table 2:** Number of procedures and relative proportions to detect prostate cancer using the four different screening strategies, using SNG-2019 as a reference strategy (*n* = 272)

Outcome	Total study population (*N* = 272)	SNG-2019	ST-3		MR		SNG-2020	
	*n*	*n*	*n*	Relative proportion to using SNG-2019	*n*	Relative proportion to using SNG-2019	*n*	Relative proportion to using SNG-2019
Performed procedures								
Performed biopsies	211	179	89	0.50 (0.41, 0.56)	123	0.69 (0.64, 0.83)	99	0.55 (0.49, 0.65)
MRI procedures	270	71	33	0.46 (0.31, 0.55)	270	3.88 (2.96, 4.35)	270	3.88 (2.96, 4.35)
Biopsy outcomes								
Benign	115	94	30	0.32 (0.21, 0.41)	58	0.62 (0.57, 0.90)	34	0.36 (0.29, 0.52)
ISUP grade 1	59	49	27	0.55 (0.41, 0.69)	35	0.71 (0.53, 0.89)	33	0.68 (0.50, 0.86)
ISUP grade ≥2	37	36	32	0.89 (0.78, 0.98)	30	0.83 (0.68, 0.97)	32	0.89 (0.76, 1.0)

CI = confidence interval; ISUP = International Society of Urological Pathology; MR = magnetic resonance imaging strategy; MRI = magnetic resonance imaging; SNG = Swedish national guideline; ST-3 = Stockholm3 strategy.

Data are *n* (%) or relative proportions (95% CI). The table shows results from the paired, screening-by-invitation, intention-to-treat analysis. SNG-2019 represent previous Swedish national guidelines without MRI and SNG-2020 represents the current Swedish national guidelines including MRI.

The use of the blood-based Stockholm3 test was shown to be beneficial to the existing Swedish national guidelines for detecting prostate cancer.

## 4 Discussion

We performed a population-based, paired, screening-by-invitation study to compare the previous and current Swedish national diagnostic guidelines for strategies based on a blood-based screening test (the Stockholm3 test) or MRI to detect csPCa in men with a PSA level of ≥3 ng/ml. When compared with the current SNGs, we found that the ST-3 strategy performs similarly for the detection of csPCa, while reducing the number of performed MRI scans by 88%, performed biopsies by 10%, and diagnosed ISUP 1 cancer cases by 18%. The national guidelines are complex to follow and at risk of underperforming compared with the ST-3 strategy. Compared with the ST-3 strategy, the previous SNG strategy detected a slightly higher number of csPCa cases (36 vs 32) while performing 100% more biopsies, detecting 118% more ISUP 1 cancers, as well as performing 115% more MRI scans. Compared with the MR strategy, the ST-3 strategy detected slightly more csPCa cases (32 vs 30), with markedly fewer MRI scans (33 vs 270) and biopsying fewer men (89 vs 123). In Supplementary Table 1, the characteristics of men with csPCa who were missed by the strategies are described; the majority of cancers missed by all strategies were graded as ISUP 2 on pathology.

Previous studies using the Stockholm3 test for the detection of prostate cancer have shown favorable results compared with screening using the PSA test only [4], [20], [21]. In these studies, the Stockholm3 test was used after a prior PSA test with a cutoff of 1.5, thus detecting csPCa in the PSA range between 1.5 and 3.0. In this study, we not only use the Stockholm3 test in another population different from the one used for the development of the test, but also utilize the commonly used cutoff level for PSA of 3.0, which puts the strategies compared on the same level. In this study, we not only compared with the PSA test, but further extended the comparison to the SNGs using PSA, free PSA, PSA density, DRE, and MRI to detect prostate cancer. The results showed that in comparison with the national guidelines (2019 and 2020), the ST-3 strategy was easy to follow, needed fewer MRI scans, and resulted in fewer biopsies being performed, as well as fewer diagnosed ISUP 1 cancers while detecting a similar number of csPCa cases. Our results thus show that the ST-3 strategy is a viable option for detecting csPCa and reducing overdetection of insignificant cancer, the number of performed biopsies, as well as the number of performed MRI examinations [22].

Studies have shown that the use of prebiopsy MRI followed by a targeted biopsy can increase the detection of csPCa in a clinical setting if used together with a systematic biopsy [12], [13], [23]. However, as a standalone procedure it can lack sensitivity for detecting csPCa, and it has been shown that MRI-targeted biopsies alone can underestimate the histological grade of some tumors [24]. We assessed a strategy using only MRI for targeted biopsies in a screening setting, and our results showed that as a standalone procedure, it performed worse than the other three strategies tested, missing seven out of 37 csPCa cases, while referring 38% more men to biopsy and detecting 30% more ISUP 1 cancers than the ST3 strategy.

There have been previous efforts to describe the Swedish diagnostic habits and the national guidelines. However, we do not know of any previous studies assessing the diagnostic performance of the strategies stated in the SNGs. Nugin et al [25] used registry data to assess how the guidelines affected prostate cancer care before and after the first publication. Their results indicated modest improvements in performance of 14 selected quality indicators in prostate cancer care after the guidelines were published. However, to make an impact on general prostate cancer care in Sweden, they recommended that feedback from prostate cancer care was needed. Another study by Nordström et al [26] using registry data from Stockholm, Sweden, showed that PSA testing was prevalent and increasing despite national recommendations against PSA screening. This is a further indication that despite the publication of guidelines in Sweden, these are generally not being followed in a systematic and clear way, calling for a more effective, organized screening program.

Among the key strengths of this study are the ongoing screening-by-invitation setting (since 2015) and well-controlled clinical processes. These clinical processes include the use of an MRI protocol suitable for high throughput as needed in a screening setting and centralized primary assessment. An external validation of the MRI assessments was performed, as well as a second evaluation in a high-volume experienced center. We also used a single, well-defined, and contemporary MRI/fusion biopsy method for disease verification and unified protocols for biopsy procedures using a centralized pathological assessment. In this study, we assessed complex diagnostic chains, and for robustness in the analysis, we aimed to decrease variability between providers throughout the study processes.

The Stockholm3 versus SNG study was performed in Värmland, Sweden, using centralized radiology and pathology, which may limit generalizability to other health care settings. In addition, the relatively small sample size in our study population is a limitation to the study. The results presented here show performance of the diagnostic strategies at one screening round; performance across multiple rounds of screening is thus unknown. The cases of csPCa missed using any of the strategies would probably have presented themselves in one of following screening rounds, as these cases are normally followed up. Since MRI-detected or Stockholm3-detected csPCa cases overlap to cover almost all cases, an effort to combine these tools may improve the selectivity. A deeper hypothesis-generating analysis of all different combinations of Stockholm3 and MRI, to try to find an optimal algorithm, would be of high interest.

## 5 Conclusions

There is a high rate of overbiopsying and overdetection of csPCa in a screening strategy aiming to find all csPCa cases. Our results indicate that the use of the Stockholm3 test for the detection of prostate cancer could be effective in the reduction of biopsies and detection of insignificant cancers, while maintaining the detection of significant cancer, compared with the previous and current SNGs, as well as a strategy based only on MRI of the prostate.

  ***Author contributions*:** Mauritz Waldén had full access to all the data in the study and takes responsibility for the integrity of the data and the accuracy of the data analysis.

*Study concept and design*: Waldén, Aldrimer, Eklund, Grönberg.

*Acquisition of data*: Waldén, Palsdottir.

*Analysis and interpretation of data*: Waldén, Aldrimer, Eklund, Grönberg, Palsdottir.

*Drafting of the manuscript*: Waldén, Lagerlöf, Eklund, Nordström, Palsdottir.

*Critical revision of the manuscript for important intellectual content*: Waldén, Aldrimer, Lagerlöf, Eklund, Grönberg, Nordström, Palsdottir.

*Statistical analysis*: Eklund, Grönberg, Palsdottir.

*Obtaining funding*: Waldén, Eklund, Grönberg.

*Administrative, technical, or material support*: Waldén, Aldrimer.

*Supervision*: Waldén, Grönberg.

*Other*: None.

  ***Financial disclosures:*** Mauritz Waldén certifies that all conflicts of interest, including specific financial interests and relationships and affiliations relevant to the subject matter or materials discussed in the manuscript (eg, employment/affiliation, grants or funding, consultancies, honoraria, stock ownership or options, expert testimony, royalties, or patents filed, received, or pending), are the following: Henrik Grönberg and Martin Eklund report four pending prostate cancer diagnostic-related patents: method for indicating a presence or nonpresence of aggressive prostate cancer (WO2013EP7425920131120), prognostic method for individuals with prostate cancer (WO2013EP7427020131120), method for indicating a presence of prostate cancer in individuals with particular characteristics (WO2018EP5247320180201), and method for indicating the presence or non-presence of prostate cancer (WO2013SE5055420130516). Henrik Grönberg reports one additional pending prostate cancer diagnostic-related patent: Method for detecting solid tumour cancer (WO2015SE5027220150311). The Karolinska Institutet collaborates with A3P Biomedical in developing the technology for the Stockholm3 test. Henrik Grönberg, Martin Eklund, and Tobias Nordström report owning shares in A3P Biomedical. All other authors declare no competing interests.

  ***Funding/Support and role of the sponsor*:** This study was funded by the County Council of Värmland, the Swedish Prostate Cancer Association, and Karolinska Institutet.

  ***Acknowledgement:*** We thank all the participants who willingly took part in this study. We thank the other members of the STHLM3 vs SNG research group (Georgios Daouacher, Tony Schönberg, Eric Le Brasseur, and Charlotta Gestblom) for their support in planning and facilitating the project. We thank Tobias Kjellberg and Anna-Carin Edström from the managing body of County Council in Värmland for their strong support in facilitating the study. We thank Dr. Rafael Grzegorek and Dr. Fred Helgesen and the nurses Petra Mossberg and Annika Hjort at C-Medical AB, Kristinehamn, Sweden, for the precision in biopsying the patients; Monika Vikner, Department of Clinical Chemistry, Central Hospital of Karlstad, Karlstad, Sweden, for keeping the laboratory network in Värmland together with all the PSA tests and the Stockholm 3 tests; Katarina Waldén, study nurse, County Council of Värmland, Karlstad, Sweden, for carefully taking care of all patient contacts and monitoring of the database; Wolfgang Krauss, radiologist, Department of Radiology, University Hospital, Örebro, Sweden, for meticulous re-evaluation of the MRI investigations; and Ola Steinberg at Karolinska Institutet, Stockholm, Sweden, for support and strong help with all logistics around the Stockholm3 laboratory issues.
