# Supplementary material for: A Head-to-head Comparison of Prostate Cancer Diagnostic Strategies Using the Stockholm3 Test, Magnetic Resonance Imaging, and Swedish National Guidelines: Results from a Prospective Population-based Screening Study
Source: Eur Urol Open Sci. 2022 Feb 18;38:32–9. doi: 10.1016/j.euros.2022.01.010 (PMC9051970; doi:10.1016/j.euros.2022.01.010)
Supplement: Supplementary data 1 [file mmc1.docx]

**Supplementary Table 1:** Characteristics of men with clinically significant cancer missed by different strategies.

| **Variable** | **Missed by 1: SNG-2019**  **n (%column)** | **Missed by 2: ST-3**  **n (%column)** | **Missed by 3: MR**  **n (%column)** | **Missed by 4: SNG-2020**  **n (%column)** |
| --- | --- | --- | --- | --- |
| All | 1 (100) | 5 (100) | 7 (100) | 5 (100) |
| **Age** |  |  |  |  |
| 49-54 | 0 (0) | 2 (40) | 0 (0) | 0 (0) |
| 55-59 | 0 (0) | 0 (0) | 2 (29) | 0 (0) |
| 60-64 | 0 (0) | 0 (0) | 0 (0) | 0 (0) |
| 65-70 | 1 (100) | 3 (60) | 5 (71) | 5 (100) |
| **Stockholm3** |  |  |  |  |
| < 11% | 1 (100) | 4 (80) | 0 (0) | 1 (20) |
| 11-29% | 0 (0) | 1 (20) | 2 (29) | 2 (40) |
| ≥ 30% | 0 (0) | 0 (0) | 5 (71) | 2 (40) |
| **PSA, ng/mL** |  |  |  |  |
| 3-4.9 | 1 (100) | 5 (100) | 3 (43) | 4 (80) |
| 5-9.9 | 0 (0) | 0 (0) | 4 (57) | 1 (20) |
| 10-19.9 | 0 (0) | 0 (0) | 0 (0) | 0 (0) |
| ≥ 20 | 0 (0) | 0 (0) | 0 (0) | 0 (0) |
| **Previous negative biopsy** |  |  |  |  |
| Yes | 0 (0) | 0 (0) | 0 (0) | 0 (0) |
| No | 1 (100) | 5 (100) | 7 (100) | 5 (100) |
| **Prostate Volume (ml)** |  |  |  |  |
| <35 | 0 (0) | 3 (60) | 5 (71) | 3 (60) |
| 35-50 | 0 (0) | 0 (0) | 2 (29) | 2 (40) |
| ≥ 50 | 1 (100) | 2 (40) | 0 (0) | 0 (0) |
| **DRE** |  |  |  |  |
| Positive | 0 (0) | 0 (0) | 1 (14) | 1 (20) |
| Negative | 1 (100) | 5 (100) | 6 (86) | 4 (80) |
| **PI-RADS** |  |  |  |  |
| 0-2 | 0 (0) | 0 (0) | 7 (100) | 4 (80) |
| 3 | 0 (0) | 1 (20) | 0 (0) | 1 (20) |
| 4-5 | 1 (100) | 4 (80) | 0 (0) | 0 (0) |
| **ISUP grade** |  |  |  |  |
| ISUP 2 small* | 0 (0) | 2 (40) | 3 (43) | 3 (60) |
| ISUP 2 large** | 1 (100) | 3 (60) | 3 (43) | 1 (20) |
| ISUP ≥ 3 | 0 (0) | 0 (0) | 1 (14) | 1 (20) |
| **PSA density (ng/mL/cc)** |  |  |  |  |
| <0.1 | 1 (100) | 2 (40) | 1 (14) | 1 (20) |
| 0.1-0.14 | 0 (0) | 2 (40) | 2 (29) | 3(60) |
| 0.15-0.19 | 0 (0) | 1 (20) | 0 (0) | 0 (0) |
| ≥ 0.20 | 0 (0) | 0 (0) | 4 (57) | 1 (20) |

* mm cancer found in biopsy < 10

** mm cancer found in biopsy ≥ 10

**Supplementary Table 2:** The parameters used in the MRI acquisition protocol.

| **Sequence type** | **TE/TR (ms)** | **Pixel spacing (mm x mm)** | **Slice thickness (mm)** | **GRAPPA** | **Averages** |
| --- | --- | --- | --- | --- | --- |
| Haste (sag) | 91/1400 | 1.2 x .12 | 6 | 2 | 1 |
| T1 tse (tra) | 12/620 | 0.9 x 0.9 | 4 | 2 | 2 |
| T2 tse (sag) | 114/6800 | 0.4 x 0.4 | 3 | 2 | 3 |
| T2 tse (tra) | 102/8790 | 0.4 x 0.4 | 3 | 2 | 2 |
| T2 tse (cor) | 102/7500 | 0.4 x 0.4 | 3 | 2 | 2 |
| Diffusion 50/500/1000(/1500) | 58/4300 | 2.2 x 2.2 | 3.5 | 2 | 1/5/10 |
